# Spatial distribution and determinants of newbornsnot receiving postnatal check-up withintwodays after birth in Ethiopia: a spatial and multilevel analysis of EDHS 2016

**DOI:** 10.1186/s12887-022-03506-9

**Published:** 2022-08-22

**Authors:** Destaye Guadie Kassie, Nega Tezera Assimamaw, Tadesse Tarik Tamir, Tewodros Getaneh Alemu, Masresha Asmare Techane, Chalachew Adugna Wubneh, Getaneh Mulualem Belay, Amare Wondim Ewuntie, Bewuketu Terefe, Adiss Bilal Muhye, Bethelihem Tigabu Tarekegn, Mohammed Seid Ali, BeletechFentie, Almaz Tefera Gonete, Berhan Tekeba, Selam Fisiha Kassa, Bogale Kassahun Desta, Amare Demsie Ayele, Melkamu Tilahun Dessie, Kendalem Asmare Atalell

**Affiliations:** 1 Department of Pediatrics and Child Health Nursing, School of Nursing, College of Medicine and Health Science, University of Gondar, Gondar, Ethiopia; 2 Department of Community Nursing, School of Nursing, College of Medicine and Health Science, University of Gondar, Gondar, Ethiopia

**Keywords:** Ethiopia, Multilevel, Newborns, Postnatal Check-up, Spatial variations

## Abstract

**Background:**

Accessibility and utilization of postnatal newborn check-ups within 2 days after delivery are critical for a child’s survival, growth, and development. However, the service delivery is not yet improved and fluctuates across the geographical locations in Ethiopia. Therefore, this study aimed to assess the spatial distribution and determinants of newborns not received postnatal check-ups within 2 days after birth in Ethiopia.

**Methods:**

A secondary data analysis of the Ethiopia Demographic and Health Survey (EDHS) 2016 was done among live births within 2 years preceding the survey. A multilevel binary logistic regression model was fitted to identify the factors associated with the outcome variable. Adjusted Odds Ratio with 95% (Confidence Interval) was calculated and used as a measure of associations and variables with a *p*-value < 0.05, were declared as statistically significant.

**Results:**

A total of 4036 live newborns in Ethiopia were included in the analysis, of whom half (51.21%) were females. The mean age of the mothers was 33+ SD 1.3, and more than 60 % (61.56%) of the mothers were not educated. The national prevalence of newborns not receiving postnatal check-ups within 2 days after birth was 84.29 (95% CI: 83.10–85.41) with significant spatial variations across the study area. Mothers who had no ANC visits were 58% higher than (AOR = 0.42(0.27–0.66) mothers who had > 4 ANC visits. Mothers who gave birth at home and others were 80% (AOR = 0.02(0.01–0.29) and 25% (AOR = 0.76(0.59–0.99), higher than mothers delivered at hospital. Rural mothers were 1.90 times higher (AOR = 1.90(1.29–2.81) than urban mothers, and mothers live in administrative regions of Afar 66% (AOR = 0.34(0.16**–**0.69), Oromia 47% (AOR = 0.53(0.30–0.91), Somali 60% (AOR = 0.40 (0.22–0.74),Benishangul 50% (AOR = 0.50 (0.27–0.92), SNNPR 67% (AOR = 0.33(0.19–0.57), Gambela 70% (AOR = 0.30 (0.16–0.56), Harari 56% (AOR = 0.44 (0.25–0.78), and Dire Dawa70% (AOR = 0.30 (0.17–0.54) were higher than Addis Abeba for not receiving postnatal checkup of new born within the first 2 days, respectively.

**Conclusions:**

Low postnatal check-up utilization remains a big challenge in Ethiopia, with significant spatial variations across regional and local levels. Spatial clustering of not receiving postanal check-ups within 2 days was observed in Afar, Oromia, Gambela, Benishangul, SNNPR, Harari, and Dire Dawa regions. Residence, ANC visits, place of delivery, and administrative regions were significantly associated with not receiving postnatal check-ups. Geographically targeted interventions to improve ANC follow-up and institutional delivery should be strengthened.

## Background

The postnatal period is a critical time for the survival of mothers and newborn babies. More than 50% of child death is within the first 2 days. Lack of postnatal care in this sensitive period might result in complications and deaths of the newborn and the mothers [1–3]. Hence, theWorld Health Organization (WHO) recommends, that all newborns and mothers should receive high-quality postnatal care within the first 42 days. For the mother who is delivered at home,the first postpartum visit must be as early as possible within 24 hours of birth. Aminimum of three postnatal health checks are suggested for both mothers and newborns on day 3 (48-72 hours), within7–14 days, and at 6 weeks [4–6]. Infant feeding, history of convulsion, fast breathing, spontaneous movement, hypothermia, hyperthermia, jaundice, cord, eyes, hygiene, and immunization status were assessed at each postnatal contact [2, 7].

Globally, 2.4 million newborns died in the first month of life, with nearly half (47%) of thedeaths having occurred in thefirst 2 days [8]. The burden of maternal and newborn mortality is high in Africa. Each year, nearly 125,000 women and 870,000 newborns die in the first week of life [9]. Sub-Sahara Africa is among the most affected area with an estimated1.16 million babies dying in the first 28 days and 850,000 in the first week of life [1, 7, 10, 11].

Even though progress has been made in reducing maternal and child mortality in Ethiopia, neonatal morality is still increasing from 29 in 2016 to 33 in 2019 [8], with significant spatial variations. Hince, understanding the spatial variations of low postnatal check-ups within 2 days after delivery is critical to come up with evidence-based geographically targeted interventions.

WHO recommends a postnatal check-up within 2 days after delivery to reduce neonatal mortality [1]. The first 2 days are the most critical time for the survival of the newborn. Unable to provide timely postnatal care at this stage will result in complications and even death. According to the 2019Ethiopian Demography Health Survey(EDHS), only 35% of newborns had received a postnatal check within 48 hrs after birth [12]. Postnatal care is the most neglected service in Ethiopia [4].

Previous studies identified different factors associated with low postnatal check-ups such as maternal age, birthorder, wealth status, delivery sites, residences, ANC follow-up, and maternal education [12].

In recent decades, efforts have been made to increase postnatal care, which in turn reduces neonatal mortality through the Sustainable Development Goal (SDG4), [13–15]. The Ethiopian government has also made a significant improvement to increase postnatal care by training and recruiting community health extension workers [2]. However, the postnatal care coverage is very low with significant spatial variations, which range from 0.1% in Afar to 74% in Addis Ababa [12]. Thus, investigating the spatial variations and determinants of low postnatal check-ups has important for the prevention and early identification of infant problemsand to meet the SDG4 goal, which aimed to reduce child mortality to 25/1000 live birth by 2030 [16]. A geographically linked data analysis using population and health facility data is important to map the low coverage of postnatal check-ups and identify inequalities in service access and provision [17, 18]. Therefore, this study aimed to investigate the spatial variations and determinants of low postnatal care coverage within two-day after birth in Ethiopia to come up with geographically targeted interventions to increase the postnatal check-ups within 2 days.

## Methods

### Study area

The study was done in Ethiopia using theEthiopian Demographic Health Survey 2016. Ethiopia is among the oldestcountry worldwide, which is located in the Horn of Africa at 3′ and 14.8″ latitude 33′ and 48′ longitude. The countryis bordered by Sudan in the west, Somalia and Djibouti in the east, Eritrea in the north, and Kenya in the south. The country has a surface area of1,112,000 km^2^. It is a rugged, landlocked country split by the Great Rift Valley, with archaeological finds dating back more than 3 million years; it’s a place of ancient culture. Among the important sites in Lalibela with its rock-cut Christian churches from the 12th–13th centuries. Aksum is the ruins of an ancient city with obelisks, tombs, Our Lady Mary of Zion church, and Gondar Fasil castles. Ethiopia is the 10th largest and the 2nd-most populous country in Africa after Nigeria. Administratively Ethiopia has ten regions (Tigray, Afar, Amhara, Benshangul, Gambela, Harari, Oromia, Somali, Southern, Nations, Nationalities, and People’s Region (SNNPR), and Sidama (a recently added and two city administrations (Addis Ababa and Dire Dawa). Concerning resident, 79.2% of the Ethiopian population lives in rural, and 43.3% is under fifteen ages [19]. Ethiopia uses a three-tier healthcare system; 1) primary healthcare systemconsists of health posts, health centers, and primary hospitals, 2) secondary healthcare consists of zonal hospitals, and 3) tertiary healthcare consists of comprehensive specialized hospitals [20].

### Study Design

Across-sectional retrospective study design was conducted to assess the geospatial variation and determinants of newborns not receiving postnatal check-ups within 2 days after birth.

### Source of data

The data used in this article were obtained from the Ethiopia Demography and Health Survey (EDHS) 2016, which was accessed at the MEASURE DHS website after securing a formal request to the MEASURE DHS program. The survey was carried out by the central statistics agency of Ethiopia, and the Ethiopia Public Health institute Ethiopia with the technical assistance provided by ICF International. The authors requested the measure DHS trough briefly stating the objectives of this analysis and access was granted to use the data on the (http://dhsprogram.com) website [21].

### Sampling and populations

The source population of this study was all mothers with newborns born in the last 2 years preceding the survey. Multi-stage stratified cluster sampling was used to select the study participants. In the first stage, 645 clusters or enumerations areas were selected randomly, and stratified into urban and rural. In the second stage, a fixed number of 28 households in each cluster was randomly selected [21]. Geographic coordinates of each survey cluster were also collected using Global Positioning System (GPS) [21]. Mothers aged 15–49 and children born within the 2 years preceding the survey in each selected household were subjected to our study.

**Fig. 1:**
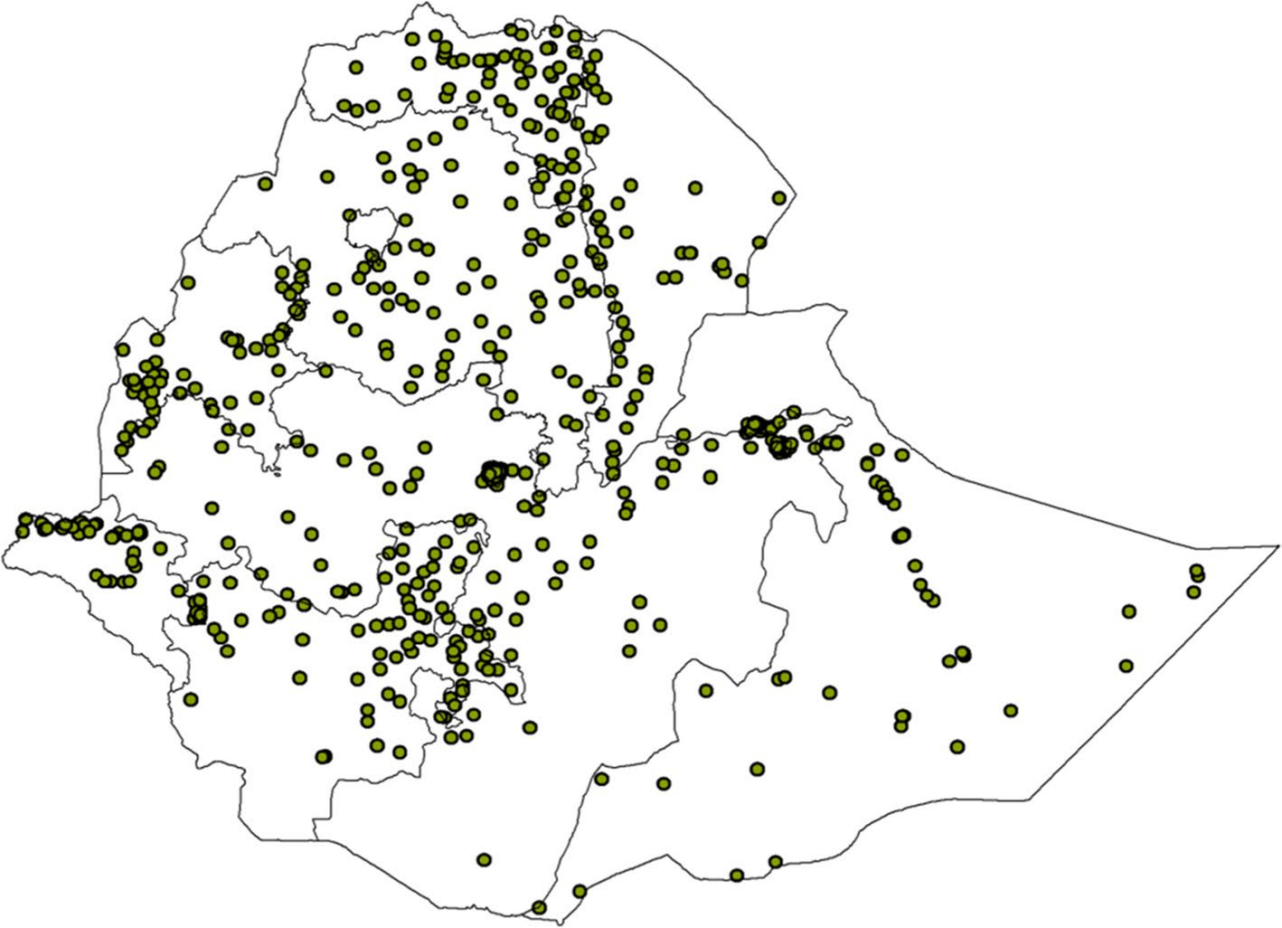
Shows the number of clusters in Ethiopia EDH S data 2016(*n* = 645 clusters)

The study was conducted among (3832 un-weighted and 4036 weighted frequency) newborns to assess the postnatal health checkups within 2 days after delivery (Fig. 1).

### Study variables

#### Outcome variable

The outcome variable for this study is postnatal check up within the first 2 days after birth. The under five data sets (KR) files, EDHS 2016 were used for this analysis, by computing the selected function of postnatal care for new born within the first 2 days after birth and relating variables. The computing variables were Cord examined (m78a) + Temperature measured (m78b) + Counseling on danger signs (m78c) + Counseling on breastfeeding (m78d) + Observation of breastfeeding (m78e). It was recorded as “No(0)” for new born not receiving a postnatal check within 2 days after birth and “yes [1]” new born receiving a postnatal check within 2 days after birth.

### Independent variables

The independent variables used in this study were as nested into; 1) individual levelfactorssuch as marital status, maternal age, religion, maternal occupation, maternal education, residence, sex of newborn, wealth index, number of ANC, place of delivery, size of the newborn at birth and number of living children inthe family. 2) Community level factors such as region,distance to the health facility, community illiteracy level, health insurance, media exposure, access to electricity, access to safe water, and community poverty. Some of the community-level factors were aggregated from the individual-level factors.

### Data management and analysis

We performed a secondary analysis of the EDHS 2016, using the Kids Records (KR) dataset. STATA version 14 and Microsoft Excel 16 were used for data cleaning and coding, and the spatial analysis, and mapping were done using ArcGIS version 10.8. Descriptive statistics such as frequency and percentage of different variables were computed and presented using texts, tables, and graphs.

After preparing the data we imported the data to ArcGIS version 10.8. Joining the outcome with the GPS data and projections of the Geographically coordinated data to the projected coordinatedata were conducted before the analysis. Spatial autocorrelation analysis was done to test whether there is spatial variation across the study area. The spatial autocorrelation test signifies whether there is clustering or dispersion of postnatal check-ups within 2 days after birth. The value of the Morans Index is standardized into Z-score. Positive Morans Index value with positive Z-score (> 1.96, *P*-value< 0.05) indicates clustering/ hot spots areas. Negative Morans Index value with a negative Z-score (<− 1.96, *P*-value1.96 (*P* < 0.05) was considered a cold spot area. GetisOrd Gi* statistic was applied to detect hotspot area or spatial clustering of newborns not receiving postnatal check-ups. Ordinary kriging interpolation was used to estimate/ predict the spatial distributions of not receiving a postnatal check within 2 days after delivery.

A multilevel binary logistic regression model was applied for each independent variable and *p*-value < 0.2 were entered into the multivariable multilevel logistic regressions model. The adjusted odds ratio was calculated and used as the measure of association between the dependant and independent variables, and variables having a *p*-value < 0.05, 95% CI were considered statistically significant.

In the EDHS data, the newborn is nested within a cluster, newborns within the same cluster were more similar to each other than within different clusters. Therefore, this violates the standard regression model assumptions, which are independence of observation and equal variance across the cluster assumptions. This implies they need to take into account between-cluster variables by using an advanced model. Therefore, a multilevel random intercept logistic regression model was fitted to estimate the association between individual-level and community-level factors and the likelihood of new-born not receiving postnatal care within 2 days after birth. Models were compared based on deviance (−2log likelihood) since the models were nested. Log-likelihood and intracellular correlation coefficient (ICC) was computed to measure the variation between clusters. The ICC indicates the degree of heterogeneity of new- born not receiving postnatal care within 2 days after birth. A multilevel binary logistic regression analysis was performed to examine the effects of individual and community level factors on newborns not receiving postnatal check-ups within 2 days after birth to identify individual and community level factors.

## Results

### Socio-demographic characteristics of the newborn

A total of 4036 weighted live birthswere included in this analysis, of whom nearly half (48.79%) of the newborns were males. The mean ages of the mother were 33+ SD 1.3 and near to one-third of the mothers (29.26%) were between the age group of 25 and 29 years. Near to 60 % of mothers, (58.97%) were unemployed, and more than 60 % (61.59%) were not educated. The majority (88.77%) of the study participants were residing in rural and nearly half (47.5%) of the mothers were in a poor wealth status (Table 1).

**Table 1:** Socio-demographic characteristics of study population in Ethiopia, EDHS 2016

Variables	Categories	Un weighted *(n = 3832*)		weighted *(n = 4036*)	
		Frequency	Percent	Frequency	Percent
Region	Tigray	409	10.67	290	7.19
	Afar	367	9.58	41	1.01
	Amhara	332	8.66	699	17.32
	Oromia	591	15.42	1827	45.28
	Somali	517	13.49	175	4.34
	Benishangul	305	7.96	43	1.05
	SNNPR	463	12.08	832	20.61
	Gambela	253	6.6	10	0.24
	Harari	226	5.9	10	0.24
	Addis Ababa	173	4.51	92	2.29
	Dire Dawa	196	5.11	17	0.42
Marital status	Never in union	3604	94.05	3801	94.17
	Married	74	1.93	80	1.97
	Widowed	30	0.78	26	0.64
	Divorced	91	2.37	84	2.09
	Others	33	0.86	45	1.12
Maternal Age	15–19	259	6.76	260	6.45
	20–24	945	24.66	918	22.76
	25–29	1098	28.65	1181	29.26
	30–34	801	20.9	868	21.51
	35–39	521	13.6	558	13.81
	40–44	165	4.31	191	4.73
	45–49	43	1.12	60	1.48
Religion	Orthodox	1109	28.94	1328	32.89
	Muslim	1946	50.78	1708	42.32
	Protestant	681	17.77	845	20.95
	Others	96	2.51	155	3.84
Maternal occupation	Not working	2377	62.03	2380	58.97
	Sale	374	9.76	447	11.08
	Agree employment	684	17.85	813	20.13
	Others	397	10.36	396	9.82
Maternal Education	No education	2307	60.2	2485	61.59
	Primary	1056	27.56	1220	30.24
	Secondary	308	8.04	230	5.69
	Higher	161	4.2	100	2.49
Residence	Urban	744	19.42	454	11.25
	Rural	3088	80.58	3581	88.75
Sex of new born	Male	1919	50.08	1969	48.79
	Female	1913	49.92	2067	51.21
Wealth index	Poor	2036	53.13	1878	47.52
	Middle	534	13.94	846	20.96
	Rich	1262	32.93	1312	32.51

*Note*: SNNPR-Southern Nations, Nationalities and Peoples Region, Occupation otters: cleric, skill & unskilled manual, service & others

### Maternal and child characteristics

More than one-third (36.04%) of mothers hadnoANC visits, and 63.64% of the mother were delivered at home. Among mothers, 53.85% had 1–3 live children in their families. The majority (96.51%) of the study participants have not used health insurance, 61.28% of the mother had a big problem reaching the health facilities, and near to 50% of the community (47.39%) were illiterate. Around 40%, (38.98%) and (72.31%) of the mother have no access to media, and electricity (Table 2).

**Table 2:** Maternal and child related factors in Ethiopia, EDHS 2016

Variables	Categories	Un weighted *(n = 3832*)		weighted *(n = 4036*)	
		Frequency	Percent	Frequency	Percent
No of ANC visit	No visit	1283	33.48	1454	36.04
	1–3 visit	1158	30.22	1247	30.92
	> = 4	1391	36.3	1333	33.04
Place of delivery	Home	2220	57.93	2528	63.64
	Government hospital	473	12.34	303	7.51
	Government health center	815	21.27	902	22.36
	others	324	8.46	302	7.48
Size of children at birth	very large	609	15.89	683	16.92
	Larger than average	503	13.13	536	13.29
	average	1581	41.26	1637	40.57
	Smaller than average	370	9.66	404	10.02
	very small	769	20.07	775	19.21
Number of living children in family	No children	40	1.04	20	0.5
	1–3	2161	56.39	2173	53.85
	4–6	1173	30.61	1281	31.74
	7–9	420	10.96	516	12.81
	10–12	38	0.99	44	1.1
Distance to health facility	Big problem	2102	54.85	2473	61.28
	Not a big problem	1730	45.15	1562	38.72
Community illiteracy level	Lowly uneducated	2060	53.76	2123	52.61
	Highly uneducated	1772	46.24	1913	47.39
Health Insurance	No	3710	96.82	3895	96.51
	yes	122	3.18	141	3.49
Media exposure	Lowly no exposed	1916	50	2463	61.02
	Highly no exposed	1916	50	1573	38.98
Access to electric city	lowly no electricity	1292	33.72	1118	27.69
	highly no electricity	2540	66.28	2918	72.31
Accesses to safe water	lowly no access water	3214	83.87	3486	86.36
	highly no access water	618	16.13	550	13.64
Community wealth index	lowly poor	1916	50	2463	61.02
	highly poor	1916	50	1573	38.98
Prevalence of newborn not receiving postnatal care	No	3230	84.29	3427	84.90
	Yes	602	15.71	609	15.10

*Note*: place of delivery others: government health center, others home, privet health facility, non-government health facility & other, ANC-Antenatal care

### The prevalence of newborns not receiving postnatal check-ups within 2 days

**Fig. 2:**
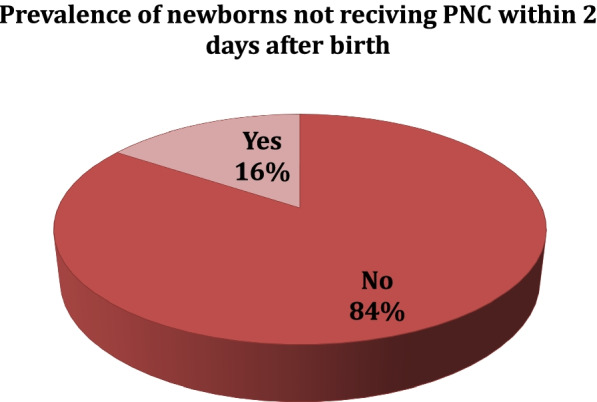
Prevalence of newborns not receiving postnatal check-up within 2 days after birth (*n* = 3, 832 un-weighted & 4036 weighted)

**Fig. 3:**
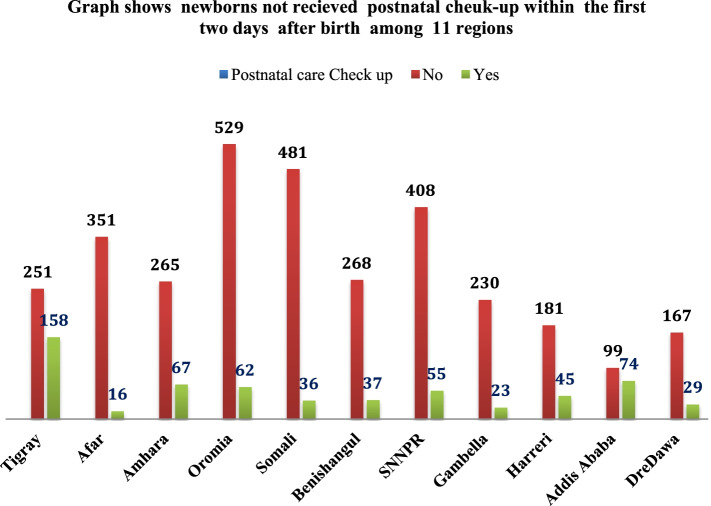
Regional distribution of the new born not receiving postnatal check up in the first 2 days after birth in Ethiopia (n = 3, 832 un-weighted & 4036 weighted)

The overall prevalence of not receiving postnatal check-ups within 2 days after birth in Ethiopia was 84% (Fig. 2)**.** The postnatal check-ups within 2 days were higher in Addis Ababa and Tigray regions, whereas, low postnatal check-ups were observed in Afar, Somali and Gambela regions (Fig. 3)**.**

## Spatial analysis result

### Spatial autocorrelation

**Fig. 4:**
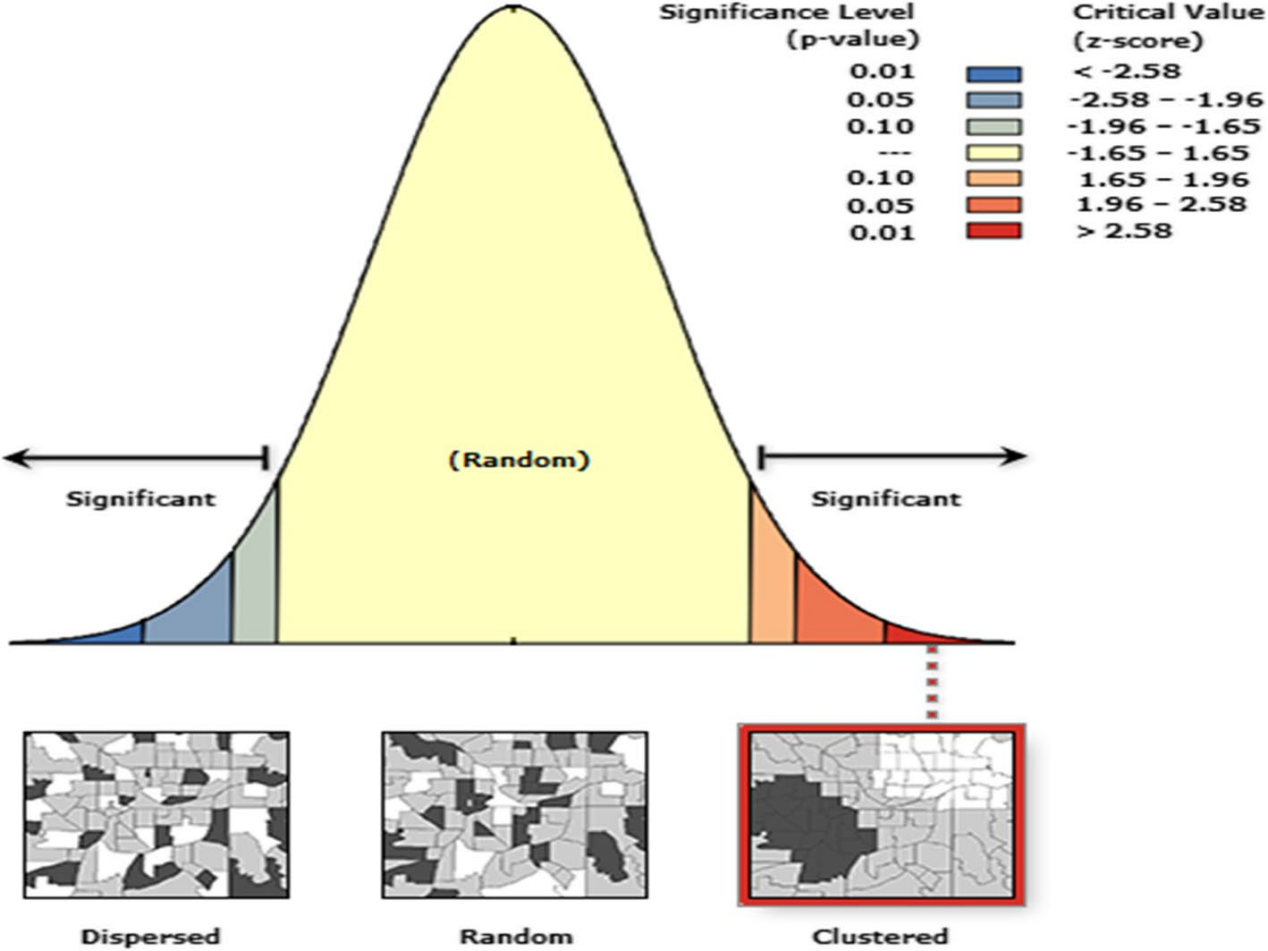
Global spatial autocorrelation of newborns not receiving postnatal check-ups within 2 days after birth in Ethiopia produced using Arc GIS version 10.8. Data EDHS 2016

The spatial autocorrelation analysis result showed that newborns not receiving a postnatal check-up within 2 days after delivery significantly varied across Ethiopia, with a Global Moran’s I value of 0.47, *p*-value< 0.0001, and z-score 7.13. This indicates that the newborn not receiving postnatal check-upswithin the first 2 days after birth in Ethiopia has spatial dependence (Fig. 4).

### Hotspot analysis of the newborns not receiving postnatal check-upsin Ethiopia

**Fig. 5:**
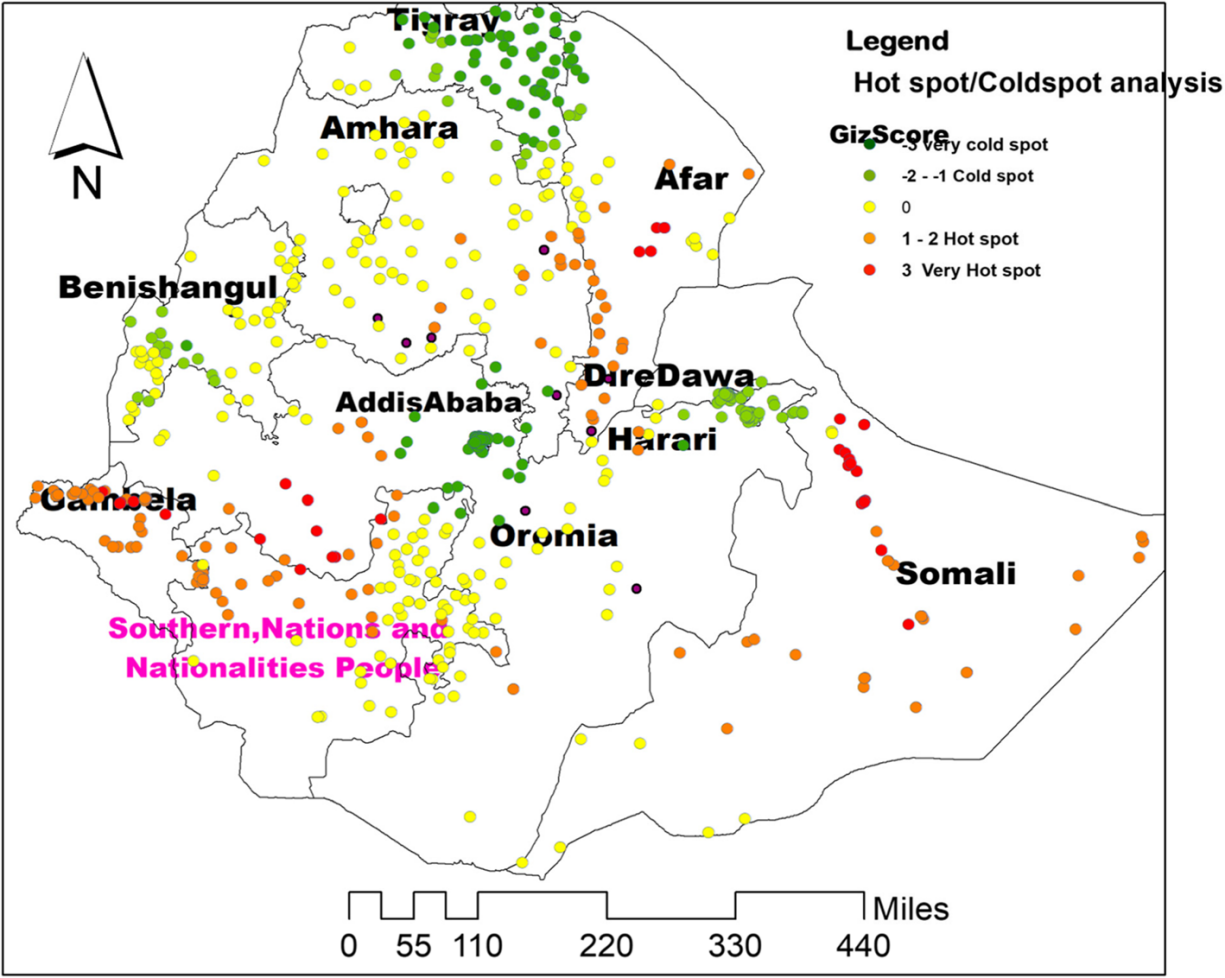
Hot spot analysis of newborns not receiving postnatal check-ups within 2 days after birth using Getis Ord Gi statistics in Ethiopia, produced using Arc GIS version 10.8

In the hotspot map, significant spatial clustering of not receiving postanal check-ups within 2 days after birth was observed in Eastern (Somali), Northeastern (Afar), Southern (Oromia and SNNPR), and Western (Gembela and Benshangul) parts of Ethiopia. Whereas, spatial dispersion of newborns not receiving postnatal check-ups was observed in Northern, Central, and Southern parts of the country specifically in regions of Tigray, Amhara, and Addis Abeba (Fig. 5).

### Satscan analysis of newborns not reciving postnatal check-up within 2 days after birth

Purely Spatial analysis scanning for clusters with high rates was applied using the Bernoulli model. A total of 287 significant clusters of newborns not receiving postnatal check-ups were specified, of which 41 and 100 were primary (most likely clusters) and secondary clusters, respectively. The primary cluster, was found in the Eastern (Somali region) of Ethiopia at 5.589269 N, 44.175032 E geospatial locations, with a relative risk of 1.47 and log-likelihood ratio (LLR) of 48.1 at a *p*-value< 0.0001. Those shows mothers who lived in this spatial window is 1.47 time at higher risk of not receiving postnatal check-up than those outside the window. Whereas the secondary clusters were honored in the Western parts of the country(Gambela and Benishangul) at 7.417914 N; and 35.317365 E geospatial locations, with relative risk and LLR of 1.29 and 31.2, respectively (Table 3).

**Table 3:** SaTScan analysis results of postnatal check-ups with 2 days after birth in Ethiopia, EDHS 2016

Cluster	Enumeration area (cluster) identified	Coordinate/Radius	Populations	Cases	RR	LLR	*P*-value
1	138, 164, 85, 358, 146, 492, 92, 490, 543, 278, 171, 198, 95, 318,77, 187, 497, 556, 520, 629, 521, 588, 553, 458, 480, 208, 214, 251,573, 239, 269, 116, 22, 394, 378, 630, 568, 33, 277, 527, 289	5.59 N, 44.18 E / 443.03 km	320	268	1.47	48.1	< 0.001
2	554, 46, 299, 526, 243, 459, 552, 168, 371, 119, 197, 326, 437, 177,325, 477, 376, 586, 555, 448, 207, 446, 219, 270, 593, 154, 265, 489,284, 114, 231, 469, 47, 221, 291, 549, 63, 417, 558, 106, 76, 337,13, 105, 343, 567, 338, 411, 432, 470, 315, 62, 603, 346, 486, 426,233, 447, 248, 69, 175, 260, 104, 227, 592, 507, 370, 306, 304, 536,435, 643, 309, 113, 406, 193, 349, 502, 70, 462, 141, 275, 126, 618,294, 161, 374, 266, 434, 142, 395, 565, 621, 450, 331, 577, 180, 466,280, 41	7.42 N, 35.32 E) / 268.40 km	642	469	1.29	31.2	< 0.001
3	4, 632, 75, 596, 440, 366, 178, 499, 205, 427, 334, 570, 348, 599, 544, 389, 368, 241, 55, 547, 191, 571, 344, 276, 332, 189, 254, 37, 249, 620, 488, 307, 135	11.85 N, 41.92 E/ 237.67 km	244	198	1.40	28.1	0.0001
4	71, 168, 552, 459, 243, 197, 526, 299, 46, 554, 437, 325, 326, 119,477, 376, 177, 207, 586, 154, 446, 448, 219, 270, 555, 337, 593, 489, 265, 284, 417, 76, 338, 231, 114, 469, 47, 221, 291, 549, 63, 13, 106, 558, 470, 105, 343, 567, 432, 411, 486, 62, 315, 603, 447, 346, 233, 426, 69, 227, 306, 260, 104, 248, 175, 507, 592, 370, 406, 113, 536, 435, 304, 141, 309, 434, 126, 502, 466, 643, 87, 450, 618, 565, 193, 142, 180	7.20 N, 35.32 E/ 260.29 km	563	412	1.3	27.2	0.0001
5	422, 34, 316, 398, 405, 468, 600, 232, 21, 518	5.84 N, 39.18E /102.80 km	93	78	1.4	13.4	0.0001
6	12, 506, 333, 476, 491, 372, 93, 122, 51, 71, 564, 245, 230, 529, 453, 441, 557, 336, 594, 25, 484, 30	9.09 N, 40.87E /116.33 km	189	142	1.3	10.8	0.010
7	235, 585, 127	13.75 N, 39.99 E /15.05 km	25	24	1.6	9.3	0.049
8	120, 24, 206, 403, 456, 38, 429	10.99 N, 38.05E /59.81 km	32	29	1.5	7.9	0.132
9	515, 615	11.07 N, 36.46 E /8.94 km	15	15	1.7	7.8	0.157
10	134, 263, 192	14.18 N, 39.98 E /27.69 km	25	23	1.6	6.9	0.357
11	566	9.46 N, 42.46 E/ 0 km	12	12	1.7	6.3	0.543
12	171	13.25 N, 40.04 E/ 0 km	11	11	1.7	5.7	0.731
13	610	9.37 N, 42.10 E/0 km	11	11	1.7	5.7	0.731
14	139	8.41 N, 38.37 E/ 0 km	10	10	1.6	5.2	0.882
15	425, 80, 551	13.35 N, 38.35E /39.54 km	29	25	1.5	5.0	0.902
16	152, 312, 327	12.69 N, 37.89 E/ 17.71 km	16	15	1.6	5.0	0.913
17	619, 26	7.47 N, 39.09 E/ 31.00 km	20	18	1.5	4.7	0.936

*RR* relative risk, *LLR* log likelihood ratio

**Fig. 6:**
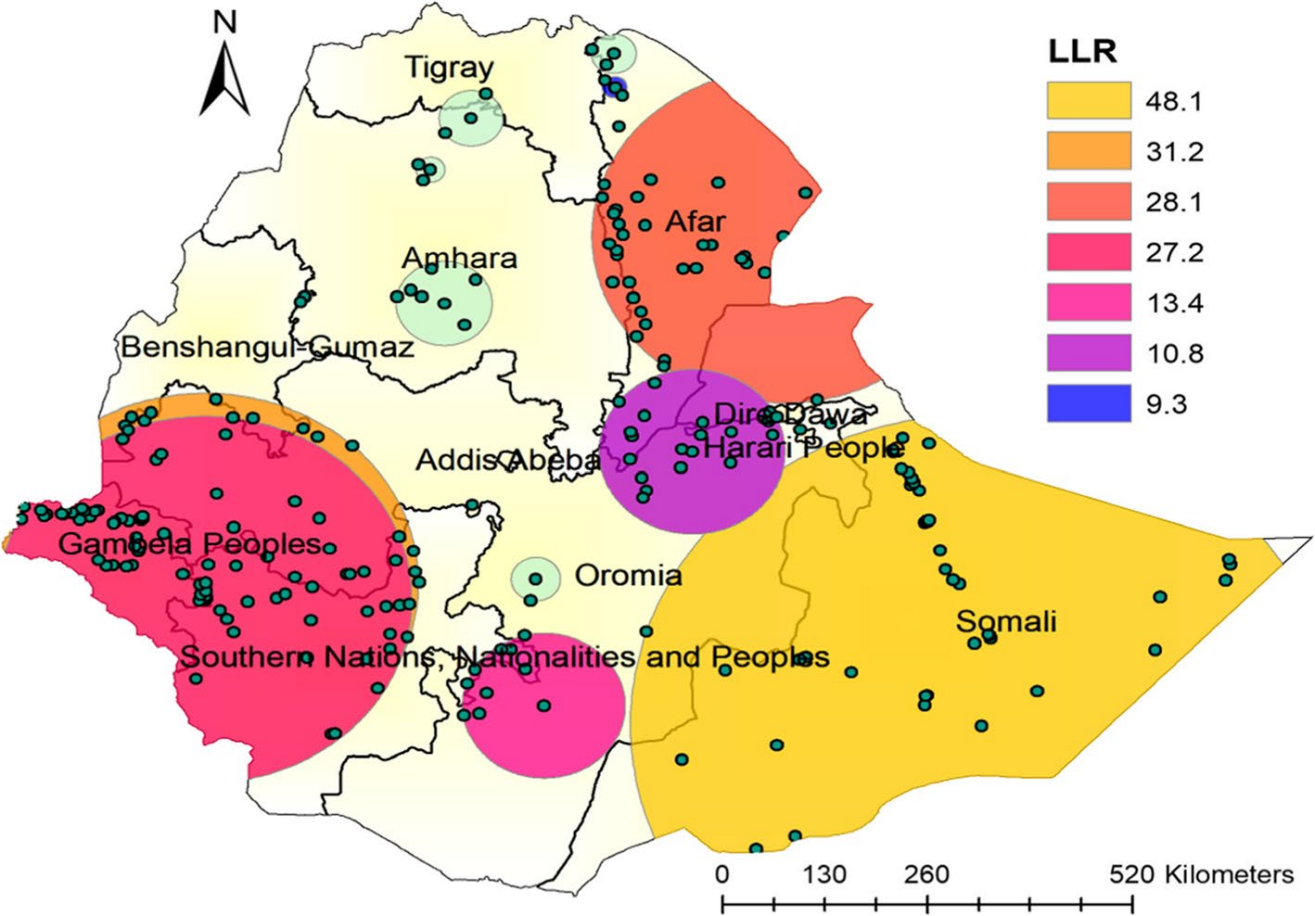
Spatial scan statistical analysis of hotspot areas of newborns not receiving postnatal check-ups within 2 days after birth in Ethiopia produced using Arc GIS version 10.8

Spatial scan statistical analysis of hotspot areas of newborns not receiving postnatal check-ups in Ethiopia, produced using Arc GIS version 10.8 (Fig. 6).

### Interpolation of a newborn not receiving a postnatal check-up within the first 2 days

**Fig. 7:**
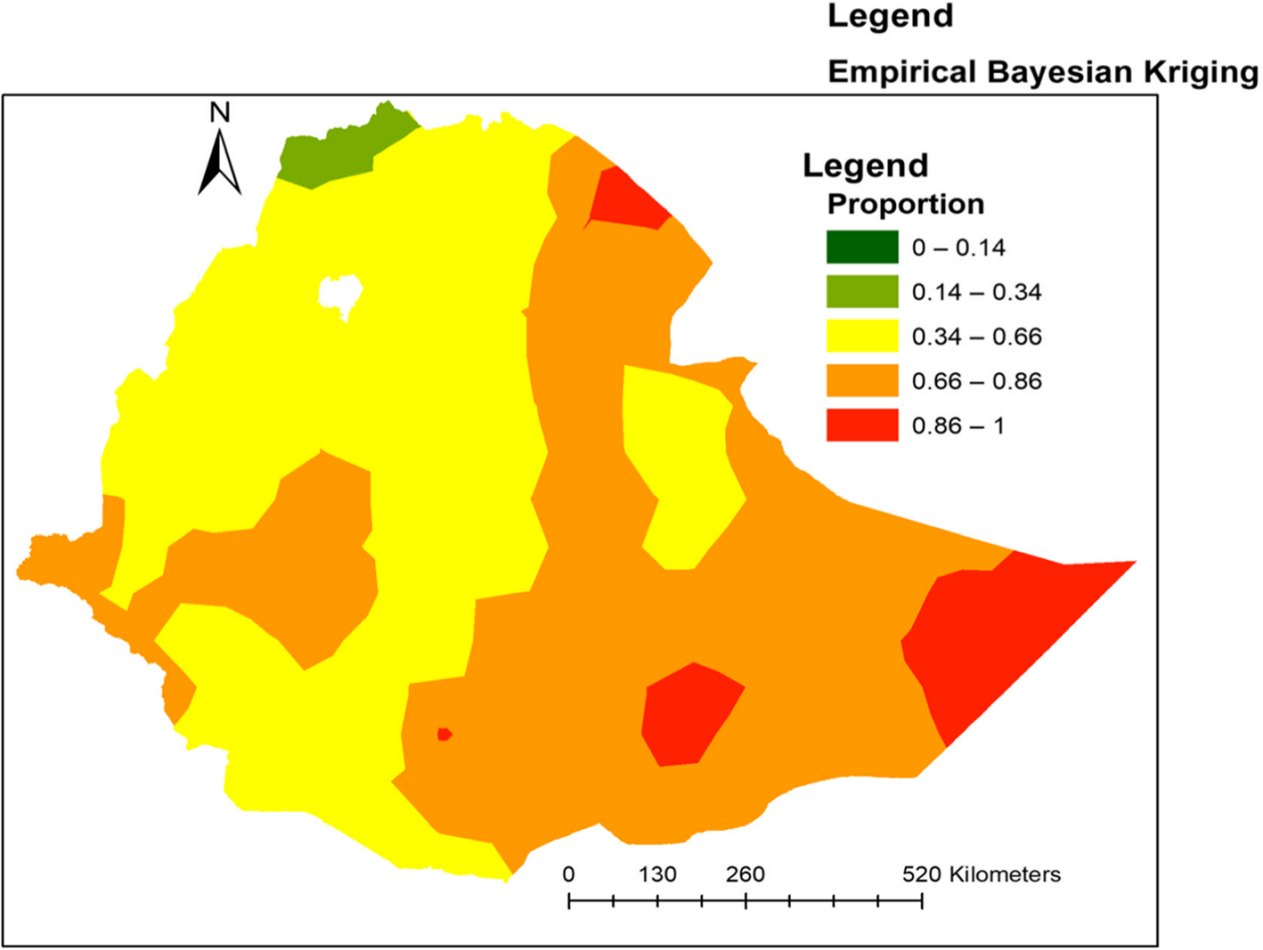
Ordinary Kriging interpolation of newborns not receiving postnatal check-ups within 2 days after birth in Ethiopia produced using Arc GIS version 10.8

Ordinary Kriging interpolation was used to predict the prevalence ofnot receiving postnatal check-ups within the first 2 days among newborns in unobserved areas. The high predicted prevalence of not receiving postnatal check-ups was observed in the Eastern, Southeastern, and Southern parts of Ethiopia. Whereas low prediction of not receiving postnatal check-upsamong newborns was observed in the Southern, Western, and Northern parts of Ethiopia, specifically in Amhara, Tigray, and Addis Ababa regions (Fig. 7)***.***

### Factors associated with newborns not receiving postnatal check-ups inEthiopia

The multilevel binary logistic regression model was the best-fitted model for our data; after LR and ICC, tests were checked. Thus, the two-level logistic regression model was fitted to obtain an unbiased result and to come up with a valid inference. Deviance was used to check the model’s fitness and a model with the lowest deviance value is the best fit model. Hence,the final model was the best-fitted model in our data, which gives the lowest deviance value. The ICC value was 0.13 (95% CI: 0.11–0.16) in the null model, which indicates that about13% of the overall variability of the newborns not receiving postnatal check-ups was presented between cluster variability, and the deviance of the null model was (−2 ×  − 1571922) = 3, 142.78. An ICC value of the individual model was 0.3442 = 34.42,95% CI: (0.17–0.71), which indicates that about 34.42% of the overall variability of the newborn, not reciving postnatal check-up was presented between cluster variability and the deviance of the individual model II was (−2 ×  − 1136.6712) = 2, 273.34, the ICC value of community level model III was 0.4582245 = 45.82, 95% CI: (0.27–0.79%) random effect whereas the deviance was (*rand*1448.7476) = 2, 8 97.50 and the multilevel model IV ICC value was 0.0902263 = 9.02, 95% CI:(0.01–0.76), and its deviance was (, *an*1101.4277) = 2, 202.86.

In this study a two level binary logistic regression model was fitted to determine the factors associated with newborns not receiving postnatal check-up. Among the individual level factors (model II); ANC visit AOR = 0.37,95%CI:(0.24–0.58), place of delivery AOR = 0.02, 95%CI: (0.01–0.03), and health insurance AOR = 0.49,95%CI:(0.30–0.80) were significantly associated with newborns not receiving postnatal check-ups; wheras, in the model III (community level factors) regions such as Afar AOR = 0.16, 95%CI:(0.08–0.34), Oromia AOR = 0.27,95%CI:(0.15–0.48), Somali AOR = 0.25, 95%CI:(0.14–0.46), Benishangul AOR = 0.37, 95%CI:(0.22–0.69), SNNPR AOR = 0.32,95%CI:(0.18–0.57), Gambela AOR = 0.24,95%CI:(0.13–0.47), Harari AOR = 0.40,95%CI:(0.22–0.72), and Dire Dawa AOR = 0.32,95%CI:(0.17–0.59), community wealth status AOR = 0.59,95%CI:(0.43–0.81),community illiteracy AOR = 0.62, 95%CI:(0.46–0.83), and media exposure AOR = 0.79,95%CI:(0.62–0.99) were significantly associated with not receiving postnatal check-up among newborns. However, in the final model (model IV) ANC visit AOR = 0.42, 95%CI: (0.27–0.66), place of delivery AOR = 0.02, 95%CI: (0.01–0.92), residence AOR = 1.90, 95% CI: (1.29–2.81) and regions such as Afar AOR = 0.34, 95%CI:(0.16–0.69), Oromia AOR = 0.53,95%CI:(0.30–0.91), Somali AOR = 0.40, 95%CI:(0.22–0.74), Benishangul AOR = 0.50, 95%CI:(0.27–0.92), SNNPR AOR = 0.33,95%CI:(0.19–0.57), Gambela AOR = 0.30, 95%CI:(0.16–0.56), Harari AOR = 0.44, 95%CI:(0.25–0.78), and Dire Dawa AOR = 0.30, 95%CI:(0.17–0.54) remains statsically significant.

In our study, the odds of not receiving postnatal check-ups within 2 days after birth was increased by 58% among mothers who have not attended ANC visits as compared to their counterparts. The odds of not receiving postnatal check-ups among mothers who delivered at home and others were increased by 80 and 25% higher than mothers who had delivered at the hospital (AOR = .0.02(0.01–0.0.29)) and (AOR = 0.76(0.59–0.99)), respectively. The majority of Ethiopian communities who have lived in different regions were significantly associated with new- borns not receiving postnatal check-ups. The odds of new borns not receiving postnatal check-up among mothers in Afar 66% (AOR = 0.34(0.16**–**0.69)), Oromia 47%(AOR = 0.53(0.30–0.91), Somali 60%(AOR = 0.40(0.22–0.74)),Benishangul 50%(AOR = 0.50(0.27–0.92), SNNPR 67%(AOR = 0.33(0.19–0.57)), Gambela 70% (AOR = 0.30(0.16–0.56), Harari 56% (AOR = 0.44(0.25–0.78)), and Dire Dawa 70% (AOR = 0.30(0.17–0.54) were higher than mothers in Addis Ababa, respectively. The odds of new born not receiving postnatal checks among mothers who had lived in rural were 1.90 times higher than in urban mothers (AOR = 1.90(1.29–2.81)) (Table 4).

**Table 4:** Multivariable multi level logistic regression analysis results as individual-level, community-level and together individual and community factors that associated with new born not receiving postnatal check up in the first 2 days after birth in Ethiopia, EDHS 2016 (*n* = 3832)

Variables	Categories	Newborns receiving PCPs within 2 days AB		Models			
		No	yes	Null Model I AOR (95%CI:)	Individual Model II AOR (95%CI:)	Community Model III AOR (95% CI:)	Over all variables Model IV AOR (95%CI:)
Maternal Age	15–19	209	50		1.06(0.68–1.66)		1.08(0.70–1.69)
	20–24	795	150		0.88(0.65–1.19)		0.88(0.65–1.19)
	25–29	929	169		1		1
	30–34	676	125		0.98(0.71–1.36)		0.94(0.68–1.30)
	35–39	441	80		1.12(0.74–1.69)		0.99(0.66–1.49)
	40–44	140	25		1.22(0.63–2.36)		1.08(0.56–2.06)
	45–49	38	5		0.54(0.13–2.35)		0.48(0.11–1.99)
Marital status	Marriage	3054	176		1		1
	Not marriage	550	52		1.13(0.76–1.68)		1.09(0.73–1.62)
Sex of new born	Male	1625	294		1		1
	Female	1605	308		1.02(0.82–1.26)		0.10(0.80–1.23)
Live children in family	No children	35	5		0.76(0.24–2.39)		0.92(0.29–2.88)
	1–3	1754	407		1		1
	4–6	1032	141		0.96(0.69–1.34)		1.04(0.75–1.44)
	7–9	374	42		1.16(0.67–1.98)		1.28(0.76–2.17)
	10–12	35	7		0.44(0.10–1.85)		0.59(0.14–2.38)
Maternal occupation	Not working	2047	330		1.05(0.75–1.47)		1.65(0.76–1.49)
	sales	298	76		1		1
	Agree employee	590	94		1.19(0.78–1.83)		0.86(0.55–1.33)
	Others	295	102		1.26(0.83–1.93)		1.21(0.80–1.84)
Maternal education	No education	2048	259		1.76(1.08–2.86)		1.57(0.96–2.59)
	Primary	844	212		1.38(0.87–2.19)		1.21(0.76–1.92)
	Secondary	228	80		1.12(0.69–1.84)		1.00(0.62–1.64)
	Higher	110	51		1		1
Wealth index	Poor	1849	187		0.95(0.71–1.28)		0.83(0.57–1.19)
	Middle	446	88		1.12(0.78–1.61)		0.93(0.63–1.36)
	Rich	935	327		1		1
No of ANC visits	No visit	1249	34		0.37(0.24–0.58)*		0.42(0.27–0.66)**
	1–3	961	197		0.97(0.76–1.24)		1.01(0.79–1.29)
	> = 4	1020	371		1		1
Place of delivery	Respondent Home	2198	22		0.02(0.01–0.03)*		0.02(0.01–0.29)**
	Health institution	296	177		1		1
	Others	736	403		0.85(0.66–1.10)		0.76(0.59–0.99)**
Children size at birth	very large	507	102		0.84(0.62–1.14)		0.89(0.66–1.20)
	Larger than average	431	72		0.76(0.54–1.07)		0.83(0.59–1.16)
	Average	1310	271		1		1
	Smaller than average	318	52		0.98(0.65–1.48)		0.96(0.64–1.43)
	Very small	664	105		1.04(0.76–1.42)		1.04(0.76–1.41)
Health insurance	No	3158	552		0.49(0.30–0.80)*		0.68(0.42–1.10)
	Yes	72	50		**1**		1
Distance to health facility	Big problem	1882	220		0.84(0.66–1.07)		0.91(0.71–1.17)
	Not big problem	1348	382		1		1
Region	Tigray	251	158			1.56(0.92–2.65)	0.94(0.58–1.51)
	Afar	351	16			0.16(0.08–0.34)*	0.34(0.16–0.69)**
	Amhara	265	67			0.65(0.36–1.18)	1.04(0.59–1.83)
	Oromia	529	62			0.27(0.15–0.48)*	0.53(0.30–0.91)**
	Somali	481	36			0.25(0.14–0.46)*	0.40(0.22–0.74)**
	Benishangul	268	37			0.37(0.20–0.69)*	0.50(0.27–0.92)**
	SNNPR	408	55			0.32(0.18–0.57)*	0.33(0.19–0.57)**
	Gambela	230	23			0.24(0.13–0.47)*	0.30(0.16–0.56)**
	Harari	181	45			0.40(0.22–0.72)*	0.44(0.25–0.78)**
	Addis Ababa	99	74			**1**	**1**
	Dire Dawa	167	29			0.32(0.17–0.59*)	0.30(0.17–0.54)**
Residence	Urban	537	207			1	1
	Rural	2693	395			1.03(0.69–1.54)	1.90(1.29–2.81)**
Community wealth level	Lowly poor	1486	430			**1**	1
	Highly poor	1744	172			0.59(0.43–0.81)*	0.94(0.66–1.33)
Community illiteracy	Lowly uneducated	1612	448			1	1
	Highly uneducated	1618	154			0.62(0.46–0.83)*	0.91(0.67–1.24)
Media exposure	No	2218	1012			0.79(0.62–0.99)*	0.98(0.75–1.29)
	Yes	302	300			**1**	1
Accesses to electric city	Lowly no electricity	977	315			**1**	1
	Highly no electricity	2253	287			0.73(0.52–1.02)	0.93(0.67–1.31)
Access to safe water	Lowly no accesses water	2673	541			1	1
	Highly no accesses water	557	61			0.90(0.64–1.25)	1.07(0.75–1.54)

*Note: ICC* Intra variability coefficient, null model (I) ICC = 0.1311856 = 13.12, 95%CI: (0.11–0.16), Deviance =  − 2 ×  − 15713922 = 3,142.78

Individual model (II) ICC = 0.3442178 = 34.42, 95% CI: (017–0.71), Deviance =  − 2 ×  − 1136.6712 = 2273.34

Community model (III) ICC = 0.4582245 = 45.82 95% CI: (0.27–0.79), Deviance =  − 2 ×  − 1448.7476 = 2, 897.50, Multilevel model (IV) ICC = 0.0902263 = 9.02, 95% CI: (0.01–0.76), Deviance =  − 2 =  − 2 ×  − 1101.4277 = 2,202.8 *SNNPR* Southern Nations Nationality Peoples’ Region, others place of delivery:, others home, privet health facility, non-government health facility & other, *PCP* Postnatal check-ups, Occupation otters: cleric, skill & unskilled manual, service & others, *AB* After birth

"*"factors associated in indivitual & community level(model II & III)

"**"factors associated in over all variable (modelIV)

## Discussion

Neonatal death is unexpectedly increased in Ethiopia in recent years, the postnatal check-up within 2 days after birth is essential to improve the survival of both the mothers and the newborns. Lack of proper care within the first 2 days leads to complications for the newborn as well as the mother. Hence, this study aimed to investigate the spatial variations and determinants of not receiving a postnatal check-up within 2 days among live births in Ethiopia [1, 22].

The overall national prevalence of newborns not receiving postnatal check-ups within 2 days after birth in Ethiopia was 84.29, 95% CI: (83.11–85.41), which is in line with the study conducted in Ethiopia 83% [23]. However, the finding of our study was slightly lower than those of the study conducted in rural Bangladesh at 90%, and in Ethiopia at 90% [5, 24]. This could be because the study conducted in Bangladesh was conducted in the rural population, which might inhibit the utilization of postnatal check-ups. The study period might be the other reason for the variations in the result. The higher non-utilization of postnatal check-ups in the previous Ethiopian study might be linked to the study period, in recent years efforts have been made to increase the maternal health service utilization through training and recruiting community health extension workers.

On the other hand, the finding of this study was higher than the study conducted in the United Kindom 44.8%, in the Southern Ethiopia 61.6% [25],Ezha district, Ethiopia 76.1% [26], Addis Abeba, Ethiopia 63% [27],and a study conducted in low and middle-income countries such as Malawi 74%, Senegal 21*%*, Mali 74.82% [28], and Nepal 66% [29–31]. The discrepancy of our study with the United Kingdom could be explained by the fact that, healthcare access, infrastructure, and awareness of the population towards the utilization of postnatal check-ups in the united kingdom [32]. The difference in studies conducted in Ethiopia might be explained by the study period and most studies conducted in Ethiopia were conducted in urban, which increases the utilization of postnatal check-ups. Furthermore, the discrepancy in the study conducted in low and middle-income countries might be explained by the low healthcare access in Ethiopia. The difference for the Nepal study might be due to the differences in socio-cultural practice, sample size and geographical differences. For instance, in the African study data were collected using house to house survey, mothers may have good health care awareness, whereas our study was conducted using a nationally representative data and 88% of the population have been lived in the rural area have little awareness of postnatal care service after birth for mothers and newborn. In Ethiopia, child birth at home is also very high, which might contribute to the low utilization of postnatal check-ups [33].

Consistent with the previous studies conducted in Ethiopia [34, 35], significant autocorrelation of not receiving postnatal check-ups were detected among newborns. Spatial clustering of early postnatal non-utilization was observed in Eastern, Northeastern, Southern, southeastern, and western parts of the country, which was supported by studies conducted in Ethiopia [35–37]. This could be linked with health care access, education, and population awareness in these areas.

According to the multilevel regression analysis results, the odds of not receiving postnatal check-ups among mothers who had no ANC visits were 58% higher than mothers who had four and above ANC visits, which is supported by studies done in Ethiopia [5, 38, 39], Tanzania [40], Uganda [41], and Kenya [41, 42]. The possible reasons might be mothers who have four and above ANC visits might get counseling regarding birth preparedness, skilled delivery, and early postnatal care, which might increase the postnatal check-up utilizations.

The odds of not receiving postnatal check-ups within 2 days after birth were higher among neonates delivered at home and others by 80 and 25% respectively as compared to mothers who gave birth at hospitals. Similar reports had been released in the previous studies conducted in Ethiopia [27, 43], Tanzania [40], and Uganda [41]. The possible reason might be mothers who gave birth in hospital might have a possibility of staying for 2 days or be counseled to have a follow-up 2 days after birth stayed and can get appropriate health care follow-up for the newborns and mothers. Whereas, mothers who gave birth at home; are culturally restricted to move out of their homes for a certain period, which reduces the postnatal care utilization.

Similar to the previous study conducted in Ethiopia [38, 44], Uganda [41], and Kenya [42], the odds of not receiving a postnatal check-up among rural mothers were 1.9 times higher as compared to their counterparts. The possible reasons might be due to the difficulty of getting healthcare access and infrastructure in rural. Education, health information, and antenatal follow-up were lower in rural, which contributes to the low utilization of postnatal check-ups.

In agreement with the previous studies conducted in Ethiopia [33–36], West African countries [45], and Pakistan [46],the odds of a newborn not receiving a postnatal check-up within 2 days after birth were higher by 66% in Afar, 47% in Oromia, 60% in Somali, 50% in Benishangul, 67% in SNNPR, 70% in Gambela, 56% in Harari and 70% in Dire Dawa as compared with Addis Abeba. The possible explanation for the low utilization of postnatal check-ups within 2 days after birth in regions other than Addis Ababa might be related to population awareness of the importance of postnatal check-ups. Moreover, socio-cultural practice, socio-economicvariation, access to healthcare facilities, availability of skilled healthcare providers for postnatal care counseling, and access to transport, that might influence the utilization of postnatal check-ups.

This study has paramount importance in reducing neonatal mortality, especially in the first 2 days by improving the postanal check-ups. High-risk areas for not receiving postnatal check-ups could be very important to design effective local, geographically targeted interventions to increase the postnatal check-ups utilizations.

### Strengeth and limitations

The strength of our study is using large sample size and a country representative data. The other strength of this study is using advanced statistical models (geo-spacial and multilevel analysis), which account the cluster variabilities. However, our study has some limitations, first, since we used secondary data analysis, we fail to incorporate some clinical variables, which might effect on the outcome variable. The second limitation of this study was we coundn’t include the paternalinformations, which might infulunce our result.

## Conclusion

Low postnatal check-up utilization remains a big challenge in Ethiopia, with significant spatial variationsacross regional and local levels. Spatial clustering of not receivingpostanal check-ups within 2 days was observed in Afar, Oromia, Gambela, Benishangul, SNNPR, Harari, and Dire Dawa regions. Residence, ANC visits, place of delivery, and administrative regions were significantly associated with not receiving postnatal check-ups. Geographically targeted interventions to improve ANC follow-up and institutional delivery should be strengthened.

## Data Availability

The corresponding author will give the data for formal requesters.

## Abbreviations

ANC: Antenatal Care
ANC: Antenatal Care Visits
CRS: Coordinate Reference System
DHS: Demographic and Health Survey
EAs: Enumeration Areas
EDHS: Ethiopia Demographic and Health Survey
GIS: Geographic Information Systems
ICC: Intra-class Correlation Coefficient
SNNPR: Southern Nations, Nationalities and Peoples Region
WHO: World Health Organization
